# Amphibian Egg Jelly as a Biocompatible Material: Physicochemical Characterization and Selective Cytotoxicity Against Melanoma Cells

**DOI:** 10.3390/polym17152046

**Published:** 2025-07-27

**Authors:** Behlul Koc-Bilican, Tugce Karaduman-Yesildal, Selay Tornaci, Demet Cansaran-Duman, Ebru Toksoy Oner, Serkan Gül, Murat Kaya

**Affiliations:** 1 Department of Molecular Biology and Genetics, Faculty of Science and Letters, Aksaray University, 68100 Aksaray, Türkiye; behlulkoc@aksaray.edu.tr (B.K.-B.); tugcekaraduman48@gmail.com (T.K.-Y.); 2 Department of Bioengineering, Faculty of Engineering, Marmara University, 34469 Istanbul, Türkiye; selaytornaci@gmail.com (S.T.); ebru.toksoy@marmara.edu.tr (E.T.O.); 3 Biotechnology Institute, Ankara University, 06135 Ankara, Türkiye; dcansaran@gmail.com; 4 Department of Biology, Faculty of Arts and Sciences, Recep Tayyip Erdogan University, 53100 Rize, Türkiye; 5 Department of Molecular Biology and Genetics, Faculty of Science and Letters, Istanbul Technical University, 34469 Istanbul, Türkiye

**Keywords:** amphibian egg jelly, anticancer, biocompatible material, melanoma, pelophylax ridibundus, real-time cell analysis

## Abstract

Extensive research on amphibians has focused on areas such as morphological and molecular taxonomy, ecology, embryology, and molecular phylogeny. However, the structure and biotechnological potential of egg jelly—which plays a protective and nutritive role for embryos—have remained largely unexplored. This study presents, for the first time, a detailed physicochemical analysis of the egg jelly of *Pelophylax ridibundus*, an amphibian species, using Fourier Transform Infrared Spectroscopy, Thermogravimetric Analyzer, X-ray Diffraction, and elemental analysis. The carbohydrate content was determined via High-Performance Liquid Chromatography analysis, and the protein content was identified using Liquid Chromatography-Tandem Mass Spectrometry analysis. Additionally, it was revealed that this jelly exhibits a significant cytotoxic effect on melanoma cells (viability < 30%) while showing no cytotoxicity on healthy dermal fibroblast cells (viability > 70%). Consequently, this non-toxic, biologically derived, and cultivable material is proposed as a promising candidate for cancer applications, paving the way for further research in the field.

## 1. Introduction

Amphibian eggs are surrounded by a thin membrane that remains viable only under moist conditions. Therefore, amphibian habitats are typically limited to areas with consistent water availability throughout most or all of the year [1]. These eggs are characterized by a gelatinous matrix known as egg jelly, which generally consists of multiple layers. This insoluble jelly matrix is a secretion product of the tubular gland cells lining the oviduct and is deposited around the eggs as they pass through the oviduct post-ovulation. Optical microscopy studies have shown that the jelly layers are morphologically simple and lack distinctive structural features [2]. Histochemical studies and chemical analyses of the amphibian egg jelly coat have revealed the presence of proteins and carbohydrates in its composition [2,3,4,5,6,7,8]. Until recently, research on amphibian eggs focused on the role of the vitelline envelope, with relatively limited attention given to the jelly layers. More recent research has predominantly examined the role of the jelly layers in fertilization and development. Studies on fertilization consistently indicate that the amphibian egg jelly layers are critical for successful fertilization and subsequent development [3,4,6,9]. These extracellular matrix layers play a crucial role in fertilization by facilitating sperm binding, triggering the acrosome reaction, preventing polyspermy, and safeguarding the developing embryo. These roles are widely conserved among the extracellular matrix layers of eggs across the animal kingdom [10,11,12].

For the first time, this study hypothesizes that the structure of amphibian egg jelly—which protects, nourishes, and supports the growth and development of embryos under harsh environmental conditions—may also exhibit significant biological activity as a potential biomaterial. This jelly acts as a multifunctional extracellular matrix that shields the embryo from external stressors, including potential aquatic pollutants, while simultaneously providing essential nutrients. After fulfilling its protective function, the jelly undergoes biodegradation and is reintegrated into the surrounding ecosystem. For the current study, the species *Pelophylax ridibundus* (*P. ridibundus*) was selected as the test organism due to its cosmopolitan distribution, ease of cultivation, and the lack of sufficient information in the literature. This species, known as the marsh frog, is a frog species belonging to the family *Ranidae* (true frogs), with an average body length of around 15 cm. Its dorsal coloration can range from greenish-gray to light or dark brown. Although studies have been conducted on the feeding habits, distribution, heavy metal accumulation in the skin due to environmental factors, changes in body size related to elevation, and age structure of this species, no research has been completed on the characterization of the egg jelly and its biological activity [13,14,15].

Considering the protective and nutritive functions of the egg jelly, particularly its potential role in shielding embryos from ultraviolet (UV) radiation, this study explores its potential applications in skin-related biomedical fields [12,16,17]. Melanoma, characterized by its high metastatic potential and aggressive nature, is responsible for approximately 90% of fatalities associated with skin cancer. Conventional therapeutic approaches, including chemotherapy, radiotherapy, and immunotherapy, have demonstrated limited effectiveness in treating metastatic melanoma, often resulting in unfavorable patient outcomes [18]. This highlights the urgent need for effective therapeutic strategies to combat this cancer type.

**Figure 1:**
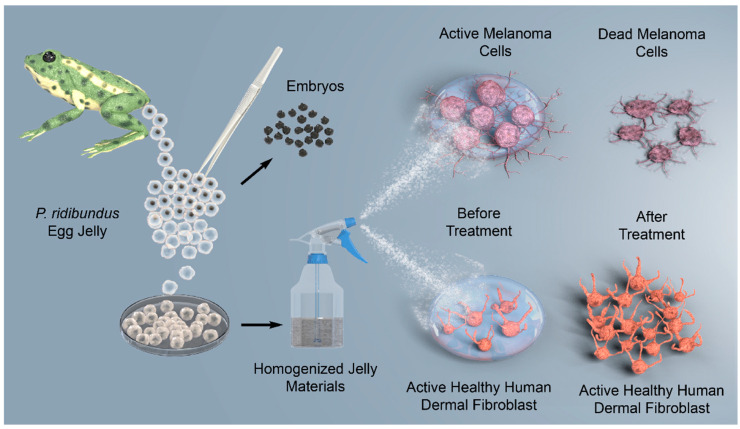
Effect of *P. ridibundus* egg jelly on melanoma and healthy dermal fibroblast cells. Egg jelly materials were homogenized and applied to melanoma cells and human dermal fibroblasts. Treatment resulted in melanoma cell death while maintaining fibroblast viability.

In this study, the previously unexplored chemical composition of *P. ridibundus* egg jelly, which protects and nourishes embryos under variable environmental conditions, was elucidated through physicochemical analyses, including FT-IR, TGA, XRD, elemental analysis, and SEM. The carbohydrate and protein contents, along with the monosaccharide composition and protein profile of the egg jelly—hypothesized to exhibit glycoprotein characteristics—were analyzed. The effects of the characterized egg jelly structure on melanoma cancer cells were investigated, and its potential toxicity to healthy human dermal fibroblasts was tested. Additionally, for the first time, the xCELLigence system, which enables real-time monitoring, was utilized to assess cancerous and healthy skin cells and to determine the effective dose for skin cancer treatment. The study is schematized in Figure 1.

## 2. Materials and Methods

### 2.1. Collection and Preparation of P. ridibundus Eggs

Egg clusters of *P. ridibundus* were collected from the Rize province and its surroundings (Salarha Valley, 41°00′42″ N, 40°33′27″ E; 572 m) during field studies. To prevent potential contamination, the collected samples were fixed in 96% ethanol and preserved for further analyses (Figure S1). The egg jelly samples were initially washed with distilled water and then with alcohol to remove any dust and impurities. Subsequently, the jelly samples were separated from the embryos and dried at room temperature (Figure S2). All procedures involving animal samples were conducted in compliance with ethical guidelines and approved by the Recep Tayyip Erdoğan University Local Ethics Committee for Animal Experiments (Decision No: 2020/45, Date: 31 December 2020).

### 2.2. Characterization of P. Ridibundus Egg Jelly

#### 2.2.1. FTIR

The IR spectra of the amphibian jelly samples were recorded using a Perkin-Elmer Spectrum Two FT-IR spectrometer (PerkinElmer, Inc., Waltham, MA, USA). Measurements were conducted in the 400–4000 cm^−1^ wavelength range with a resolution of 8 cm^−1^.

#### 2.2.2. XRD

XRD analysis, based on the characteristic scattering of X-rays due to the unique atomic arrangements of crystalline phases, was performed at 40 kV and 30 mA within a 2θ range of 5–45°. Peak intensities in the spectra of the *P. ridibundus* jelly samples were identified, and the percentage crystallinity of the jelly was calculated using the following equation (Equation (1)):

(1) CrI100=[(I110−Iam)/I110]×100

*CrI*100 = percentage crystallinity, *I*110 = maximum intensity at 2θ ≈ 20°, *I_am_* = maximum intensity of the amorphous peak at 2θ ≈ 13°.

#### 2.2.3. TGA

The thermal stability, as well as the water and ash content of the amphibian jelly samples, were analyzed using an Exstar-TG/DTA 7300 thermogravimetric analyzer (Hitachi High-Tech Corp., Tokyo, Japan). Thermal stability was assessed by heating samples from 30 °C to 1000 °C at 10 °C/min under a constant nitrogen flow.

#### 2.2.4. SEM-EDX

Three-dimensional images of the surface and inner layers of the amphibian egg jelly samples were recorded at 100×−1000× magnification using a Fei Quanta FEG 250 SEM (FEI Company, Hillsboro, OR, USA) operated at 5 kV. Prior to imaging, the lyophilized jelly samples were coated with gold/palladium using a Cressington sputter coater 108 auto (Ted Pella, Inc., Redding, CA, USA). The EDX spectra of the jelly samples were recorded using an Ametek Edax Octane Pro EDX detector (EDAX Inc., Mahwah, NJ, USA).

#### 2.2.5. Elemental Analysis

The percentage content of C, N, H, O, and S elements in the amphibian egg jelly structure was determined with high precision using a Flash 2000 elemental analyzer (Thermo Fisher Scientific Inc., Waltham, MA, USA).

#### 2.2.6. Determination of Carbohydrate Concentration and Profile

The phenol-sulfuric acid assay, as described by Dubois, was employed to quantify the carbohydrate concentration [19]. A 1 mL sample of amphibian jelly was mixed with 25 μL of 80% phenol and 2.5 mL of H_2_SO_4_, and the mixture was incubated in a water bath at 30 °C for 15 min. The prepared samples were measured at a wavelength of 490 nm using a UV spectrophotometer. Monosaccharide composition was performed using HPLC (Agilent 1260, Midland, ON, Canada) (see the Supporting Information for details).

#### 2.2.7. Determination of Protein Concentration and Profile

Proteins were precipitated using the TCA-acetone protocol to ensure compatibility with the HPLC working conditions. The precipitated protein was washed with 200 µL of acetone and centrifuged under the same conditions. To remove any residual acetone, the protein pellet was allowed to stand at room temperature for 5 min. The pellet was then vortexed until fully dissolved in 50 mM ammonium bicarbonate ((NH_4_)HCO_3,_ AmBic, Witney, UK) and 0.1% formic acid (HCOOH, FA). Protein concentrations, after cleaning with the TCA-acetone precipitation method, were measured using the Bradford Assay [20]. Prior to LC-MS/MS analysis, the general profiles of the prepared samples were examined using sodium dodecyl sulfate-polyacrylamide gel electrophoresis (SDS-PAGE). The proteins obtained from the egg jelly were subjected to tryptic digestion for mass spectrometry analysis. For this, a trypsin digestion kit (In-solution tryptic digestion kit, Thermo Fisher) was used, following the kit’s protocol. The concentration of the resulting peptides was evaluated using the Qubit 4.0 device.

Peptide separation was performed using a Dionex Ultimate 3000 Series RSLC nano pump (Thermo Fisher Scientific Inc., Waltham, MA, USA). The samples passing through the column with continuous nano-flow were ionized and subjected to LC-MS/MS analysis. Top10 MS/MS analysis was performed for each parent ion. Protein identification was conducted using Proteome Discoverer 2.2 software, based on data obtained from LC-MS/MS analysis. The data were cross-referenced with organism-specific information from the Uniprot/SwissProt database. Functional and biological classification of the proteins was performed according to Gene Ontology data in the Proteome Discoverer 2.2 extension (see the Supporting Information for all details of the method).

### 2.3. Cell Culture Studies

The A-375 human skin melanoma cancer cell line (ATCC No. CRL-1619) was cultured in high-glucose DMEM (Sigma-Aldrich, Catalog No. D6429; St. Louis, MO, USA) supplemented with 10% Fetal Bovine Serum and 1% penicillin/streptomycin. The healthy human dermal fibroblast cell line (PCS-201-012) was cultured using the Bullet Kit (Lonza, Catalog No. CC-3249). Both cell lines were maintained in an incubator at 37 °C with 5% CO_2_.

#### 2.3.1. MTT Assay

The MTT assay was performed to indirectly evaluate cell proliferation and/or cytotoxicity. This analysis aimed to determine the effects of different amphibian jelly concentrations on cell viability and calculate IC_50_ values. Jelly concentrations (10:1 mg mL^−1^, with subsequent 1:2 serial dilutions up to 7 concentrations) were prepared based on literature guidelines and incubated with the cancer and normal cell lines for specific periods (24, 48, and 72 h). For the assay, 1 × 10^4^ cells per well were seeded in 96-well plates containing 100 µL medium for each cell line. At the end of the specified periods, the medium was removed, and the cells were treated with the jelly at predetermined doses and time points. Following incubation, cells were treated with 10 µL of MTT reagent (5 mg mL^−1^) in 100 µL fresh medium for 3 h at 37 °C. The resulting formazan crystals were subsequently solubilized using 100 µL DMSO after careful removal of the reaction mixture. The absorbance was then recorded at 570 nm using a microplate reader, and cell viability was determined based on Equation (2). Wells containing only medium (without gel) were used as the control group, and cell-free medium was used as the blank.

% Viable cell = ((*A_sample_* − *A_blank_*)/(*A_control_* − *A_blank_*)) × 100 (2)

#### 2.3.2. Real-Time Cell Analysis (RTCA) Using xCELLigence

A-375 and healthy human dermal fibroblast cells were seeded at a concentration of 1 × 10^4^ cells per well in 100 µL medium in 16-well plates (ACEA Biosciences) and monitored using the xCELLigence system. Once the cells entered the logarithmic growth phase, 10 mg mL^−1^ amphibian jelly and its serial dilutions were added to the wells. A control group without treatment was included for comparative analysis. The cells were treated with the jelly material for 96 h, and their proliferation was monitored every 15 min in an incubator at 37 °C with 5% CO_2_. The xCELLigence RTCA system recorded impedance-based Cell Index (CI) values every 15 min over a 96 h period, resulting in high-resolution, real-time monitoring of cell proliferation dynamics. All experiments were conducted in triplicate (*n* = 3) for both melanoma (A375) and healthy dermal fibroblast (HDF) cell lines. The RTCA Software Lite (version 2.0) automatically calculates IC_50_ values using sigmoidal dose–response models, incorporating built-in statistical algorithms that include standard deviation estimations for each dose–response curve. This provides a statistically robust and unbiased determination of IC_50_ values without manual intervention. Comparisons between treatment groups and controls were evaluated using one-way ANOVA followed by Tukey’s post hoc test. A *p*-value < 0.05 was considered statistically significant. This label-free method evaluated the anti-proliferative effect of the amphibian jelly on the melanoma cell line and its cytotoxicity on the human dermal fibroblast cell line.

#### 2.3.3. Statistical Analysis

All cell-based experiments were conducted in triplicate (*n* = 3) using both melanoma (A375) and HDF cell lines. Cell viability data obtained from the MTT assay were analyzed using GraphPad Prism software version 9 (GraphPad Software^®^, San Diego, CA, USA) and expressed as mean ± standard deviation (SD). Statistical differences between treatment groups and controls were evaluated using one-way ANOVA followed by Tukey’s post hoc test, with a 95% confidence interval. A *p*-value < 0.05 was considered statistically significant.

## 3. Results and Discussion

### 3.1. FTIR

**Figure 2:**
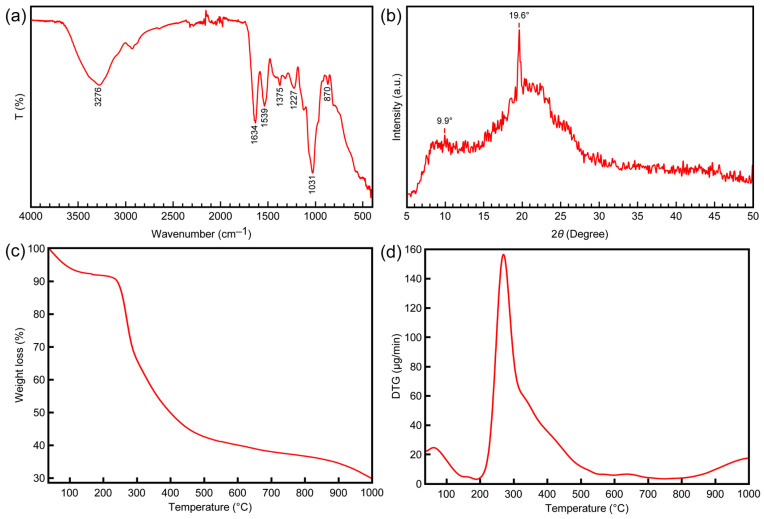
Characterization results of the egg gel sample: (**a**) FT-IR spectrum, (**b**) XRD pattern, (**c**) TGA curve, and (**d**) DTG curve.

FT-IR analysis was performed on amphibian egg jelly to measure the vibrational frequencies of various bonds in the molecules and to gather information about their functional groups. The recorded FT-IR spectrum for *P. ridibundus* egg jelly is presented in Figure 2a. Amide absorption bands are characteristic for proteins [21] and they arise from the polypeptide backbone, being sensitive to the protein’s structure. These bands typically appear at 1600–1700 cm^−1^ for Amide I, 1504–1582 cm^−1^ for Amide II, and 1200–1300 cm^−1^ for Amide III [21]. For the *P. ridibundus* egg jelly, the Amide I band was observed at 1634 cm^−1^, attributed to C=O stretching, which is a guide for identifying the secondary structure of proteins [22]. The Amide II band appeared at 1539 cm^−1^, resulting from N–H bending and C–N stretching vibrations [23]. The Amide III band, observed at 1227 cm^−1^, originates from in-plane N–H bending vibrations combined with C–N stretching vibrations [23]. All these bands confirm the presence of proteins in the amphibian egg jelly structure. The Amide I band recorded between 1610–1640 cm^−1^ is characteristic of an antiparallel β-sheet [21,24,25,26]. The observed Amide I band at 1634 cm^−1^ supports the presence of β-sheets in the secondary structure of the proteins in the amphibian egg jelly. Previous studies have demonstrated that β-sheet structures impart strong mechanical properties to materials [27,28]. This finding supports the notion that amphibian egg jelly preserves its structural integrity even in polluted aquatic environments and harsh conditions, thereby safeguarding the embryo. Additionally, the band observed at 1031 cm^−1^ represents the polysaccharide rings in the structure. This peak corresponds to ring vibrations overlapping with the stretching vibrations of glycosidic bonds and the C–O–C linkage [29]. The broad band recorded at 3276 cm^−1^ is attributed to the intermolecular hydrogen bonding of absorbed water’s O–H groups combined with N–H stretching vibrations [30].

### 3.2. XRD

The crystal structure of *P. ridibundus* egg jelly was determined using XRD analysis, with the results shown in Figure 2b. The analysis revealed two distinct sharp peaks at 9.9° and 19.6°. In the literature, structural proteins such as collagen, keratin, and silk have characteristic peaks reported in the ranges of 7.8–10.7° and 19.6–21.8° [31,32]. The peaks observed for the amphibian egg jelly align with these characteristic peaks, confirming its composition of proteins and carbohydrates. While the two peaks are characteristic of proteins, the peak near 19° is attributed to the polysaccharide content [33,34]. The peak at 19.6° was particularly intense due to the combined contributions of protein and polysaccharide components. The crystallinity of *P. ridibundus* egg jelly was calculated to be 70.23%, which is relatively high compared to several other natural materials. In a previous study, the crystallinity of chitin extracted from silkworm pupae was reported to be 47%, from shrimp shells 54%, and from beetle larvae cuticles 56% [35]. In another study, nanocellulosic samples isolated from *S. oleoides* exhibited a crystallinity of 66.5%, while cellulose derived from tomato peels showed a crystallinity of 69% [36,37]. Additionally, the crystallinity of moth cocoon protein was reported to be 62% in a separate study [38]. Increased crystallinity correlates with greater structural stability and mechanical strength [39,40]. These findings support the notion that the amphibian egg jelly is a robust biomaterial capable of protecting the embryo in harsh environmental conditions and polluted aquatic ecosystems.

### 3.3. TGA

The TG/DTG analysis results for *P. ridibundus* egg jelly are presented in Figure 2c,d. The thermogram clearly indicates two distinct degradation peaks. The first peak, observed between 0–150 °C, corresponds to the removal of entrapped water molecules within the structure [41]. The maximum degradation rate (DTG_max_) for this peak was recorded at 56 °C, with a mass loss of 8.2%. The second peak, observed between 200–1000 °C, exhibited a DTG_max_ of 267 °C, with a mass loss of 62.5%, attributed to protein-polysaccharide degradation. This degradation temperature range shows remarkable agreement with the thermal behavior documented for structural proteins including collagen, keratin, and fibroin [42,43,44]. The relatively lower DTG_max_ observed for the amphibian egg jelly compared to other structural proteins is attributed to the carbohydrate content of the material. This degradation involves peptide bond cleavage and the breakdown of side chain groups of amino acid residues [45,46,47]. The high thermal stability of this natural material is noteworthy. Such stability significantly contributes to the ability of amphibian egg jelly to maintain its structural integrity in harsh aquatic environments, thereby protecting the embryo.

### 3.4. SEM

**Figure 3:**
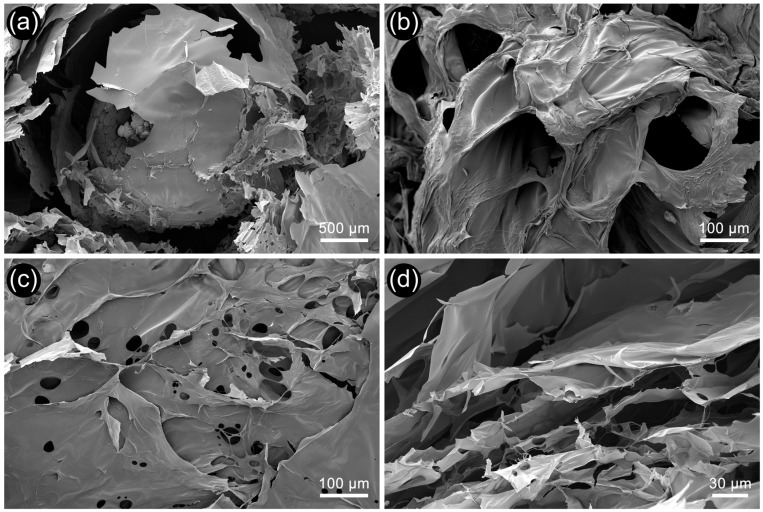
Microscopic analysis of the egg gel sample: (**a**) Embryo inside the egg gel, (**b**) SEM images of the egg gel dried at room temperature, (**c**) Surface morphology of the lyophilized egg gel, and (**d**) Cross-sectional view of the lyophilized egg gel.

SEM analysis is an essential tool for examining the three-dimensional surface morphology of biomaterials. Using this analysis, the surface structures of amphibian egg jelly samples were clearly observed. Both compact samples dried at room temperature (Figure 3a,b) and samples dried using a lyophilizer to preserve their three-dimensional structure (Figure 3c,d) were analyzed via SEM. In both analyses, the SEM images revealed that *P. ridibundus* egg jelly is composed of layers. Previous studies have demonstrated that amphibian egg jellies are multilayered, with their structure varying across species [2,48]. The SEM images of dried jelly layers for *P. ridibundus* indicated no distinctive morphology; the structure appeared smooth, with occasional large pores between the layers.

The layering of the jelly around the embryo occurs during its passage through the oviduct [2,3]. In our study, the layered structure surrounding the embryo was clearly visualized after freeze-drying with a lyophilizer (Figure 3c). As reported in the literature, the jelly layers consist of a network of fibrillar structures, which was also evident in SEM images [48]. In freeze-dried samples where the three-dimensional structure was preserved, the layers were interconnected through pores, as shown in the SEM images (Figure 3d).

### 3.5. Elemental Analysis and EDX

Elemental analysis was performed to determine the elemental composition and profile of the amphibian egg jelly sample. To corroborate the findings and provide a qualitative analysis, EDX analysis was also conducted. The results of both analyses are presented in Table 1.

The elemental profile of *P. ridibundus* egg jelly was found to consist of nitrogen, carbon, hydrogen, and sulfur. According to the analyses, the elemental composition of the jelly was determined as 5.46% nitrogen, 36.84% carbon, 5.36% hydrogen, and 1.33% sulfur. These elements were confirmed via the EDAX detector in the EDX analysis. The quantities of elements identified in the profile are comparable to those reported for structural proteins such as sericin, fibroin, and the structural carbohydrate chitosan [49,50,51]. Variations in carbon and nitrogen percentages can arise due to differences in the protein’s position and chemical structure [23].

### 3.6. Determination and Profiling of Carbohydrate Content

**Figure 4:**
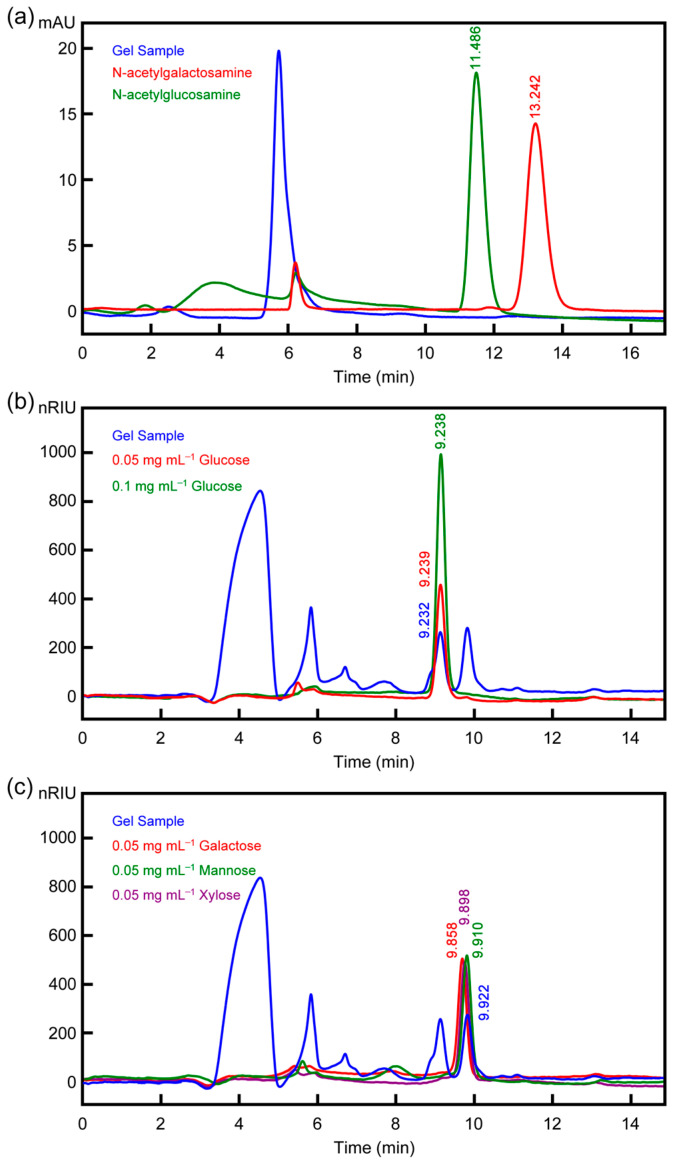
HPLC chromatograms of the egg jelly sample and standard monosaccharides. (**a**) Comparison with N-acetylgalactosamine (red) and N-acetylglucosamine (green). (**b**) Comparison with glucose standards (0.05 mg mL^−1^ in red, 0.1 mg mL^−1^ in green). (**c**) Comparison with galactose (red), mannose (green), and xylose (purple), each at 0.05 mg mL^−1^. Retention times of the standards are marked for peak identification.

Standard glucose solutions were prepared in the concentration range of 0.5–10 µg mL^−1^. The color of the samples varied from light yellow to dark yellow depending on the carbohydrate content. These solutions were analyzed at 490 nm using a UV spectrophotometer, and a standard calibration curve was plotted based on the absorbance values obtained, as shown in Figure S3. Using the Dubois method, the carbohydrate concentration was determined from the experimental data and the equation derived from the standard calibration graph. The results indicated that the carbohydrate concentration of *P. ridibundus* egg jelly was 96.43 ± 0.024 µg mL^−1^. In the HPLC analysis, efforts were made to detect glucose, mannose, xylose, fucose, galactose, galactosamine, and glucosamine sugars, which are reported in the literature to be present in amphibian egg jelly (Figure 4).

The chromatogram showed the presence of glucose, galactose, mannose, and xylose sugars. These findings align with previously reported sugars in amphibian egg jelly [4,7,52]. Additionally, the free glucose content was determined to be 0.28 ± 0.01 mg mL^−1^. The inability to distinguish galactose, mannose, and xylose separately is attributed to the overlapping retention times of these sugars, as evident from Figure 4c. Literature data confirm the presence of these sugars, and the overlapping retention times of the sample support these findings [4,7,52].

### 3.7. Determination and Profiling of Protein Content

The crude protein content was determined using the Kjeldahl method, where the nitrogen content (±N) obtained from the analysis was converted using a factor of 6.25. The analysis revealed that *P. ridibundus* egg jelly contained 26.65% protein (*w*/*w*). Additionally, Bradford analysis was performed to determine the total protein concentration (Figure S4). The analysis indicated that 1 µL of *P. ridibundus* egg jelly contained 2.4 µg of total protein. To examine the overall profile and sample purity, proteins were resolved on 12% SDS polyacrylamide gels (SDS-PAGE). Samples were loaded at increasing concentrations, and gels were stained with Coomassie dye to verify protein concentrations. The resulting bands demonstrated high quality and cleanliness of the isolated protein samples. The isolated samples were subjected to LC-MS/MS analysis. The proteins identified using the Uniprot/Swissprot database are listed in Table 2.

One of the proteins identified in *P. ridibundus* egg jelly was keratins, which are structural proteins predominantly found in epithelial cells [53]. Keratins belong to the intermediate filament protein superfamily, which is crucial for maintaining mechanical stability and integrity between cells [54]. Keratins also play roles in regulatory functions, such as intracellular signaling pathways involved in wound healing. The specific keratin identified, Keratin Type I protein (xk81b1), was also observed in *Xenopus laevis*, where it is noted to play a key role during embryonic and tadpole developmental stages [55]. During amphibian metamorphosis, nearly all tissues and organs undergo remodeling, with keratin playing a significant role in these processes [56]. Another protein identified in *P. ridibundus* egg jelly was Elongation Factor 1-alpha. This protein acts as a selective regulator of growth factors, with low levels found in amphibian oocytes but active expression in somatic cells [57,58]. Bombesin, another protein identified, is an active peptide previously purified from amphibian skin [59]. This 14-amino-acid peptide has numerous effects, including the stimulation of hypothermia, DNA replication, and the release of various gastrointestinal hormones [60,61,62,63]. Additionally, it participates in appetite suppression, controls smooth muscle contractility and glandular secretions (both exocrine and endocrine), mediates thermoregulation and blood pressure maintenance, and influences sucrose metabolism and cellular proliferation [64]. Additionally, lectin has been reported to possess the potential to be developed into anticancer drugs due to its ability to induce apoptosis and autophagy in cancer cells [65].

Other proteins identified in *P. ridibundus* egg jelly include: KAT8 regulatory NSL complex subunit 1-like protein, which has regulatory acetyltransferase activity [66], Elongator complex protein 2, a multiprotein complex with conserved elongation factor activity [67]. Protein FAM214A, a member of the growth factor-associated protein family [68]. Tudor domain–containing proteins, which play crucial roles in epigenetics, gene expression, and RNA regulation [69]. Arpin protein regulates a crucial role in cytoskeletal dynamics, contributing to both its structural organization and stability [70]. Another significant protein identified was the sialic acid–binding lectin. Lectins are multivalent carbohydrate–binding proteins capable of agglutinating normal cells (e.g., erythrocytes), cancer cells, and microorganisms [71]. Specifically, the identified protein exhibited selective binding to sialic glycoproteins, leading to the preferential agglutination of cancer cells [72]. Moreover, this lectin inhibits tumor growth in vivo through a macrophage system targeting tumor cells [73]. Its activities include protection against pathogenic microorganisms [74]. Considering these findings, it can be predicted that the valuable proteins in its composition confer strong biological activity to the egg jelly.

### 3.8. Cell Culture Studies

#### 3.8.1. MTT Assay Results

**Figure 5:**
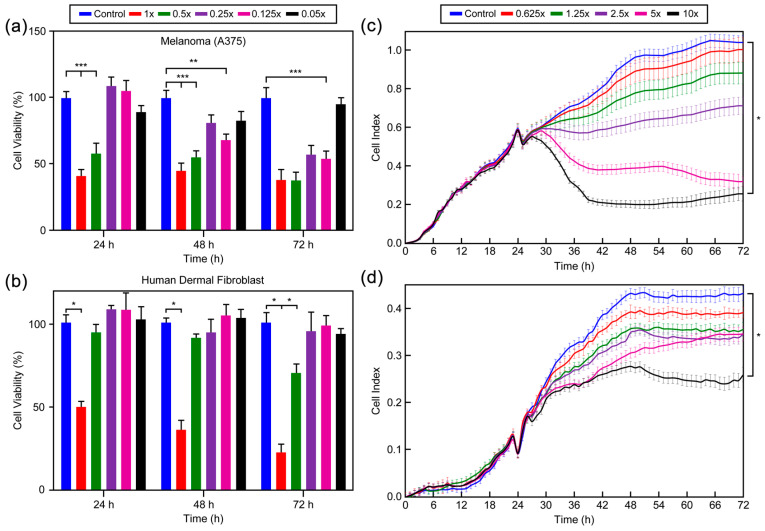
Cell viability (%) results of the MTT assay: (**a**) Melanoma (A375) cells (** *p* < 0.01; *** *p* < 0.001; *n* = 3) and (**b**) Human dermal fibroblasts (* *p* < 0.05; *n* = 3). Cell index measurements from xCELLigence analysis: (**c**) Melanoma (A375) cells (* *p* < 0.05; *n* = 3) and (**d**) Human dermal fibroblasts (* *p* < 0.05; *n* = 3) (control group refers to wells containing medium only, without gel).

The biological activity of *P. ridibundus* egg jelly against melanoma cancer cells and its cytotoxicity on healthy human dermal fibroblast cells were evaluated using the MTT assay. A colorimetric method was employed to assess the effect of the egg jelly on melanoma cancer cell (A375) proliferation and its cytotoxicity on healthy HDF cells by measuring the percentage of surviving cells. The MTT assay evaluates cell viability based on the enzymatic reduction of tetrazolium salts to formazan by living cells. After dissolving the crystals with DMSO, the color intensity was measured using an ELISA reader. The results demonstrated that amphibian egg jelly exhibited a dose- and time-dependent antiproliferative effect on melanoma cancer cells. Furthermore, the egg jelly showed no cytotoxic effects on healthy human dermal fibroblast cells, as confirmed by MTT analysis. The time, dose, and cell viability results from the MTT assay are presented in Figure 5a,b.

Serial dilutions were prepared from a 10:1 mg mL^−1^ concentration of amphibian egg jelly and applied to the cells. Based on the results, concentrations of 5 mg mL^−1^, 2.5 mg mL^−1^, and 1.25 mg mL^−1^ were identified as inhibitory for melanoma cancer cells while being non-toxic to healthy dermal fibroblast cells. This study demonstrates that *P. ridibundus* egg jelly selectively inhibits melanoma cancer cells without exhibiting toxicity to healthy cells.

#### 3.8.2. xCELLigence Real-Time Cell Analysis Results

The xCELLigence system was employed to evaluate the effect of *P. ridibundus* egg jelly on melanoma cancer cell (A375) proliferation and its cytotoxicity on healthy HDF cells. The system monitors cellular dynamics in real time by measuring electrical impedance changes using an e-plate. Doses determined from the MTT assay (1.25, 2.5, and 5 mg mL^−1^) were tested on melanoma cancer and healthy dermal fibroblast cells using the xCELLigence system, providing simultaneous measurements. The time, dose, and cell viability results from xCELLigence analysis are shown in Figure 5c,d. Our findings demonstrated that a 5 mg mL^−1^ concentration of amphibian egg jelly showed no cytotoxicity in healthy cells (viability > 70%) while exhibiting significant anticancer activity against melanoma cells (A375, viability < 30%). This concentration was identified as the selective dose. This study evaluated the comparative cytotoxic effects of *P. ridibundus* egg jelly extract on melanoma (A375) and HDF cells. The results showed that a concentration of 5 mg mL^−1^ led to the inhibition of more than 70% of melanoma cells, while maintaining the viability of more than 70% of healthy HDF cells. This suggests a biologically selective effect whereby the extract targets cancerous cells preferentially, with minimal toxicity to normal cells. Although IC_50_ values are typically employed to quantify pharmacological efficacy, this study’s primary objective was not solely to determine the average inhibitory concentration. Rather, the aim was to identify a selective and biologically safe dose that effectively targets melanoma cells while preserving the viability of healthy cells. Therefore, the 5 mg mL^−1^ dose corresponding approximately to an IC_70_ level in melanoma cells was considered a selective and functionally relevant dose. This approach aligns with current trends in anticancer biomaterial research, where selective cytotoxicity is a crucial criterion for therapeutic potential and safety profiling. These findings highlight the potential of *P. ridibundus* egg jelly as a novel biomaterial for melanoma cancer treatment and its promise for biotechnological and biomedical applications.

## 4. Conclusions

This study is the first to comprehensively characterize the egg jelly of *P. ridibundus* from a materials science perspective and evaluate its potential as a biomaterial with anticancer properties. The jelly’s unique structure, composed primarily of glycoproteins and carbohydrates, provides mechanical support, protection against environmental stressors, and nutrient supply to the embryo. FT-IR analysis confirmed the presence of β-sheet proteins, which contribute to the jelly’s mechanical integrity. Thermal stability tests revealed degradation at 267 °C, indicating a robust structure. XRD analysis demonstrated a high crystallinity of 70.23%, further supporting the material’s exceptional stability. Elemental and EDX analyses confirmed the presence of C, N, H, O, and trace amounts of S, while SEM imaging highlighted its layered morphology surrounding the embryo. Biochemical analyses identified glucose, galactose, mannose, and xylose as the major monosaccharides, while protein profiling via LC-MS/MS detected 12 proteins, including sialic acid-binding lectins known for their tumor-inhibiting properties. Based on these findings, the biological activity of *P. ridibundus* egg jelly against melanoma cancer can be attributed to the strong activity of its protein components. Functional assays demonstrated that the egg jelly exhibits significant anticancer activity against melanoma (A375) cells at a selective dose of 5 mg mL^−1^, reducing cell viability to less than 30% while maintaining over 70% viability in HDF cells. These findings reveal *P. ridibundus* egg jelly as a non-toxic, biocompatible material with selective anticancer activity, offering a promising avenue for melanoma treatment, while the need to elucidate its mechanisms of action inspires further research into its biomedical applications.

## Figures and Tables

**Table 1:** Elemental and EDX analysis results of egg gel sample.

Elemental Analysis	% N	% C	% H	% S
	5.46	36.84	5.36	1.33
EDX Analysis	Weight (%)			
	C K	N K	O K	S K
	51.00	7.66	34.11	5.48

**Table 2:** Proteins identified through research in the UniProt/Swiss-Prot database.

Master	Accession Number	Description
Protein	P02537	Keratin-3, type I cytoskeletal 51 kDa OS = *Xenopus laevis* OX = 8355 PE = 2 SV = 1
Protein	P05782	Keratin, type I cytoskeletal 47 kDa (Fragment) OS = *Xenopus laevi*s OX = 8355 GN = xk81b1 PE = 3 SV = 2
Protein	P08776	Keratin, type II cytoskeletal 8 OS = *Xenopus laevis* OX = 8355 PE = 2 SV = 1
Protein	P13549	Elongation factor 1-alpha, somatic form OS = *Xenopus laevis* OX = 8355 GN = eef1as PE = 2 SV = 1
Protein	P16878	Keratin, type II cytoskeletal OS = *Xenopus laevis* OX = 8355 PE = 2 SV = 2
Protein	P18839	Sialic acid-binding lectin OS = *Rana japonica* OX = 8402 PE = 1 SV = 3
Protein	P21591	Bombesin OS = *Bombina orientalis* OX = 8346 PE = 2 SV = 1
Protein	Q0IHW6	KAT8 regulatory NSL complex subunit 1-like protein OS = *Xenopus tropicalis* OX = 8364 GN = kansl1l PE = 2 SV = 1
Protein	Q5EBD9	Elongator complex protein 2 OS = *Xenopus tropicalis* OX = 8364 GN = elp2 PE = 2 SV = 1
Protein	Q5FW46	Protein FAM214A OS = *Xenopus tropicalis* OX = 8364 GN = fam214a PE = 2 SV = 1
Protein	Q5M7P8	Tudor a protein 7 OS = *Xenopus tropicalis* OX = 8364 GN = tdrd7 PE = 2 SV = 1
Protein	Q66IV5	Arpin OS = *Xenopus laevis* OX = 8355 GN = arpin PE = 2 SV = 1

## Data Availability

All data are available in the manuscript or electronic Supplementary Material.

## Supplementary Materials

The following supporting information can be downloaded at: https://www.mdpi.com/article/10.3390/polym17152046/s1, Figure S1: *P. ridibundus*, the streambed where it was collected, egg clutches, and microscopic images; Figure S2: Preparation and drying process of amphibian egg gel samples; Figure S3: Standard calibration curve obtained for absorbance values; Figure S4: SDS-PAGE analysis result of the egg gel sample; Table S1: Gradients of mobile phases applied to the analytical column in nHPLC.
