# Supplementary material for: Amphibian Egg Jelly as a Biocompatible Material: Physicochemical Characterization and Selective Cytotoxicity Against Melanoma Cells
Source: Polymers (Basel). 2025 Jul 27;17(15):2046. doi: 10.3390/polym17152046 (PMC12349566; doi:10.3390/polym17152046)
Supplement: Supplementary file 1 [file polymers-17-02046-s001.zip › polymers-3741749-supplementary.pdf]

Supporting Information for

Amphibian Egg Jelly as a Biocompatible Material: Physicochemical Characterization and Selective Cytotoxicity Against Melanoma Cells

Behlul Koc-Bilican[a*], Tugce Karaduman-Yesildal[a], Selay Tornacı[b], Demet Cansaran-Duman[c], Ebru Toksoy Oner[b], Serkan Gül[d*], Murat Kaya[e*]

[a] Department of Molecular Biology and Genetics, Faculty of Science and Letters, Aksaray University, 68100 Aksaray, Türkiye

[b] Department of Bioengineering, Faculty of Engineering, Marmara University, Istanbul, Türkiye

[c] Biotechnology Institute, Ankara University, 06135, Ankara, Türkiye

[d] Department of Biology, Faculty of Arts and Sciences, Recep Tayyip Erdogan University, Rize 53100, Türkiye

[e] Department of Molecular Biology and Genetics, Faculty of Science and Letters, Istanbul Technical University, Istanbul 34469, Türkiye

Corresponding Authors

Serkan Gül

Department of Biology, Faculty of Arts and Sciences, Recep Tayyip Erdogan University, Rize, Türkiye.
E-Mail: serkan.gul@erdogan.edu.tr

Murat Kaya

Department of Molecular Biology and Genetics, Faculty of Science and Letters, Istanbul Technical University, Istanbul 34469, Türkiye.
E-Mail: muratkaya3806@yahoo.com

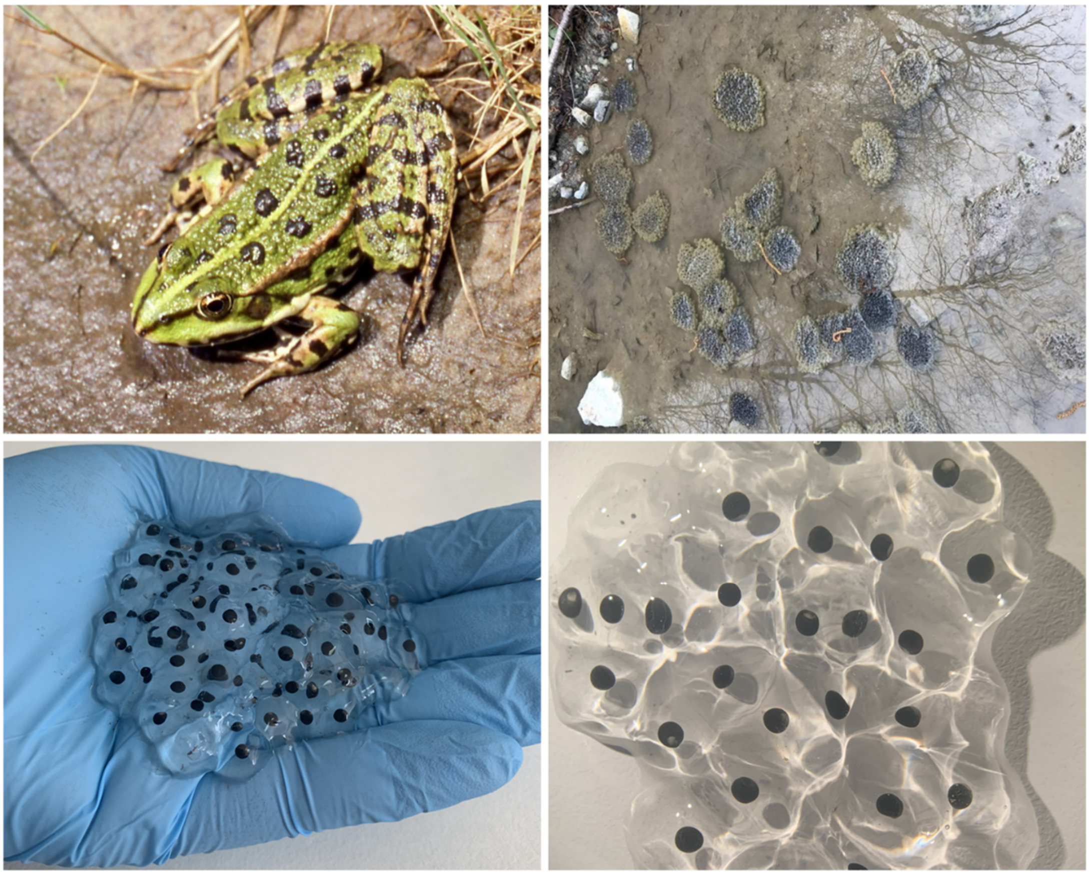

Figure S1. *P. ridibundus,* the streambed where it was collected, egg clutches, and microscopic images.

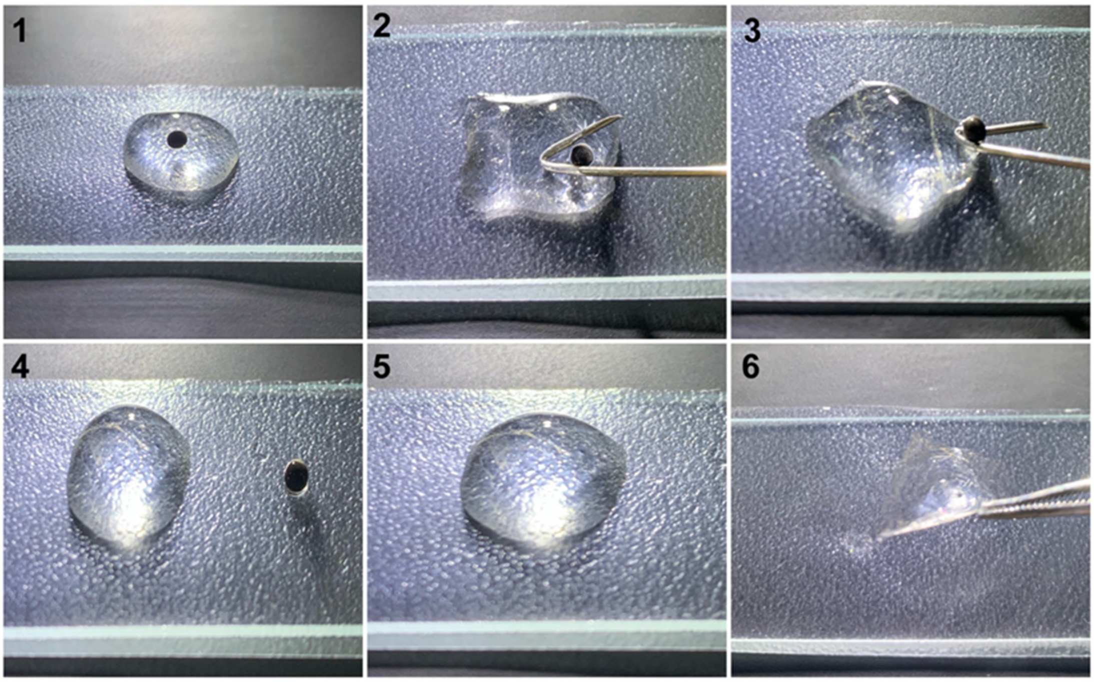

Figure S2. Preparation and drying process of amphibian egg gel samples.

Determination of Carbohydrate Profile

A total of 0.1 g of dried frog jelly was homogenized in methanol (HPLC grade) using a vortex and subsequently filtered through a 0.45 μm membrane filter (Minisart, Sartorius Stedim Biotech, Germany). For glucose analysis, the jelly sample was detected using a refractive index detector (RID). A Hi-plex H column (8 μm, 4x250 mm, Agilent, USA) was used for chromatographic separation. Injection volumes were set at 10 μL, and extract concentrations were adjusted to 100 mg/mL. The analysis was conducted at 30 °C. Samples were prepared in distilled water, with injection volumes maintained at 10 μL. The mobile phase consisted of an isocratic flow of water at a flow rate of 0.6 mL/min. A calibration curve was established using standard glucose solutions at concentrations of 0.025, 0.05, 0.1, 0.5, and 1 mg/mL in distilled water (R^2=0.9998). The limit of quantification (LOQ) for glucose was determined to be 0.015 mg/mL.

For N-acetylgalactosamine and N-acetylglucosamine analysis, the jelly sample was detected using a photodiode array (PDA) detector operating at 195-900 nm, with measurements taken at 210 nm. A MetaCarb 87H column (7.8x300 mm, Agilent, USA) was used for chromatographic separation. The mobile phase consisted of an isocratic flow of 0.008N H2SO4 at a flow rate of 0.6 mL/min. Standard solutions of N-acetylgalactosamine and N-acetylglucosamine at concentrations of 0.025, 0.05, 0.1, 0.5, and 1 mg/mL were prepared in distilled water, and calibration curves were established with R^2 values of 0.9985 and 0.9977, respectively. The LOQ values for N-acetylgalactosamine and N-acetylglucosamine were determined to be 0.019 mg/mL and 0.017 mg/mL, respectively.

For galactose, mannose, and xylose analysis, the jelly sample was detected using RID. A Hi-plex H column (8 μm, 4x250 mm, Agilent, USA) was employed for chromatographic separation. The mobile phase consisted of an isocratic flow of water at a flow rate of 0.6 mL/min. Standard solutions of galactose, mannose, and xylose at concentrations of 0.025, 0.05, 0.1, 0.5, and 1 mg/mL were prepared in distilled water, and calibration curves were established with R^2 values of 0.9975, 0.9997, and 0.9987, respectively. The LOQ values for galactose, mannose, and xylose were determined to be 0.015 mg/mL, 0.018 mg/mL, and 0.016 mg/mL, respectively.

Determination of Protein and Amino Acid Profile

To ensure the suitability of proteins for HPLC analysis, protein precipitation was performed using the TCA-Acetone protocol. One-fourth of the frog jelly sample was added to the TCA samples and incubated on ice for approximately 10 minutes. The prepared samples were then centrifuged at 14,000 ×g for 5 minutes to obtain precipitated proteins. The resulting protein pellet was washed with 200 μL of acetone and centrifuged under the same conditions. To eliminate residual acetone, the pellet was left at room temperature for 5 minutes. The pellet was then vortexed until dissolved in 50 mM ammonium bicarbonate (AmBic) and 0.1% formic acid (FA).

Protein concentrations, purified via the TCA-Acetone precipitation method, were determined using the Bradford Assay. The protein obtained from 1 μL of egg jelly was diluted with 19 μL of distilled water. Then, 1 mL of Bradford Reagent (1X, Bio-Rad, USA) was added, mixed, and incubated in the dark for 5 minutes. The protein concentrations of the prepared samples were measured at 595 nm using a Nanodrop spectrophotometer (Thermo Scientific, USA). Calculations were performed by comparing the results with a bovine serum albumin (BSA) standard curve prepared at 595 nm. Each measurement was conducted in triplicate, and the mean value of these measurements was recorded as the final protein concentration.

Before proceeding with Liquid Chromatography-Mass Spectrometry (LC-MS/MS) analysis, the general profiles of the prepared samples were examined using the sodium dodecyl sulfate–polyacrylamide gel electrophoresis (SDS-PAGE) method. The obtained samples were analyzed in 12% SDS polyacrylamide gels to assess their general profiles and purity. The samples were loaded in increasing concentrations, and the gels were stained with Coomassie dye to verify protein concentrations, evaluate band quality, and assess sample purity.

Proteins were separated based on their molecular weights using the SDS-PAGE method. The SDS gel consisted of two parts: a resolving gel and a stacking gel. After preparing 50 mL of resolving gel, it was poured between thick and thin glass plates with a 1 mm spacer, and 300 μL of isopropanol (IPA) was added to smooth the gel surface. Once polymerized, the resolving gel was washed with distilled water to remove IPA and dried. The stacking gel was then poured over the polymerized resolving gel, and a Teflon comb with 1 mm-wide wells was placed on the glass plates to create sample wells.

The electrophoresis of SDS gels was carried out by placing glass plates into Tetracell electrophoresis tanks (BioRad, USA). To prepare the system for electrophoresis, 1X SDS-PAGE running buffer was added to the electrophoresis tanks. Protein extracts to be loaded onto the gel were mixed with 1X loading buffer, which was prepared from 6X stock solution containing 0.5 M Tris-HCl (pH 6.8), glycerol (99.7%), 10% SDS, β-mercaptoethanol (0.5% w/v), bromophenol blue, and dH_2O. The samples were then incubated in a water bath at 95 °C for 4 minutes to ensure complete protein denaturation. After incubation, the samples were placed on ice, centrifuged, and loaded into the wells of the gel.

Electrophoresis was performed at 180 V for 60 minutes. Following separation, the gel was removed from the glass plates, fixed with 40% methanol and 10% acetic acid, and subsequently stained with Coomassie blue for visualization. For mass spectrometry analysis, proteins obtained from the egg gel were subjected to tryptic digestion. An in-solution tryptic digestion kit (Thermo Fisher) was used, following the manufacturer's protocol. The protocol included reduction with DTT (dithiothreitol) at 95 °C for 5 minutes, alkylation with iodoacetamide at room temperature in the dark for 20 minutes, and digestion with trypsin at 37 °C for 5 h or at 30 °C overnight. The concentration of the resulting peptides was assessed using a Qubit 4.0 fluorometer.

Peptide separation was performed using a Dionex Ultimate 3000 Series RSLC nano-pump (Thermo Scientific, USA). This pump was equipped with Ultimate 3000 Series TC C-3000RS (Thermo Scientific, USA) column compartments and was connected to a Dionex UltiMate 3000 Series RS (Thermo Scientific, USA) autosampler unit. The software used was Xcalibur 4.0 (Thermo Scientific, USA). The samples were loaded onto a trapping column containing C18 material with dimensions of 5 mm × 300 μm i.d., 5 μm, 100 Å at a flow rate of 5 μL/min using a solution containing 0.05% (v/v) trifluoroacetic acid and 1% acetonitrile. After the trapping column, the samples were transferred to an analytical column, Acclaim PepMap RSLC (Thermo Scientific, USA), packed with C18 material with dimensions of 15 cm × 75 μm and a particle size of 2 μm, 100 Å. Peptide elution was performed using two different mobile phases (A and B). Mobile phase A contained 0.1% (v/v) formic acid prepared in HPLC-grade water, while mobile phase B contained 0.1% (v/v) acetonitrile prepared in HPLC-grade water. Peptides were separated over 130 minutes at a flow rate of 0.3 μL/min and sent to a mass spectrometer (Thermo Q-Exactive). The gradient steps applied for elution are given in Table S1.

Table S1. Gradients of mobile phases applied to the analytical column in nHPLC.

Application Range (min)	Increase in Mobile Phase B Percentage	Applied Gradient Type
0-45	6-20	Lineer
45-75	40	Lineer
75-90	90	Lineer
90-120	90	Sabit
120-125	6	Rapid decline
125-130	94	Washing with phase A

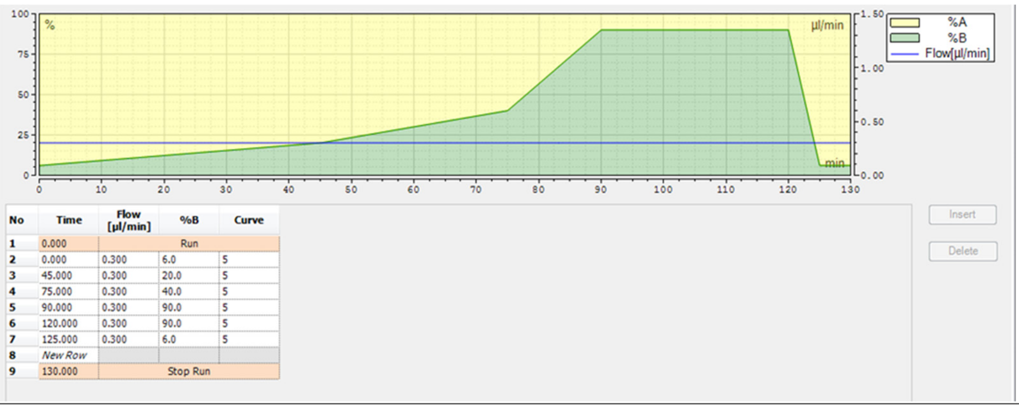

In each analysis, a 2 µL injection was performed at a temperature of 40 °C. Peptide ionization was achieved in positive ion detection mode using a heated electrospray ionization (HESI) source. The instrument parameters were set as follows: spray voltage of +2.3 kV, capillary temperature of 300 °C, with sheet gas and auxiliary gas flow rates maintained at approximately 50 and 30 units, respectively. The S and RF lens levels were adjusted to 50. Samples passing through the column under continuous nano-flow conditions were ionized and subjected to LC-MS/MS analysis. A TOP10 MS/MS analysis was conducted for each precursor ion.

Protein identification from LC-MS/MS data was performed using Proteome Discoverer 2.2 (Thermo Scientific, USA) software. The parameters applied for this analysis included a peptide mass tolerance of 10 ppm, an MS/MS mass tolerance of 0.2 Da, a mass accuracy of 2 ppm, and an allowance of one missed cleavage site. The minimum peptide length was set to six amino acids. Fixed modifications included carbamidomethylation, while variable modifications included methionine oxidation and asparagine deamination. A minimum of two peptides per protein was required for identification, and the data were searched against organism-specific datasets available in the UniProt/Swiss-Prot database. Functional and biological classification of proteins was performed based on Gene Ontology (GO) data using the Proteome Discoverer 2.2 extension.

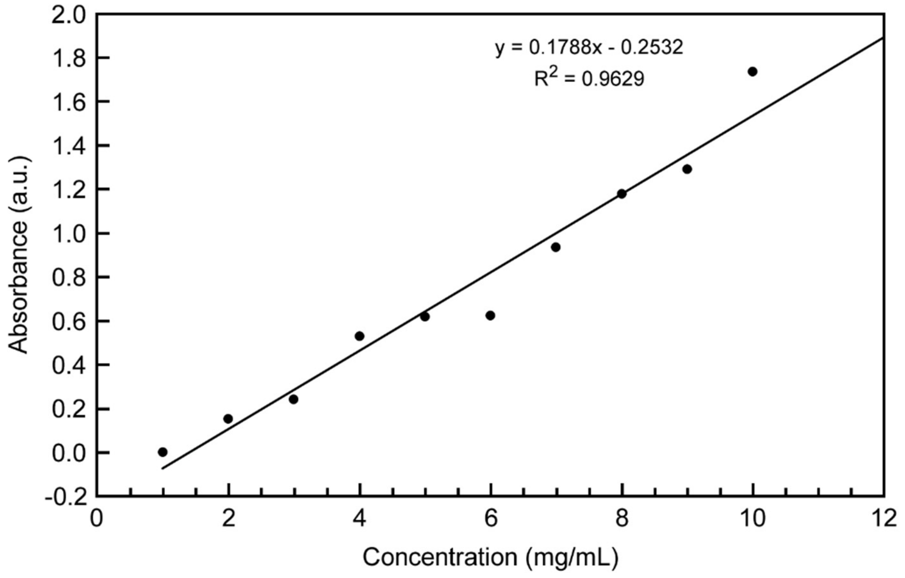

Figure S3. Standard calibration curve obtained for absorbance values.

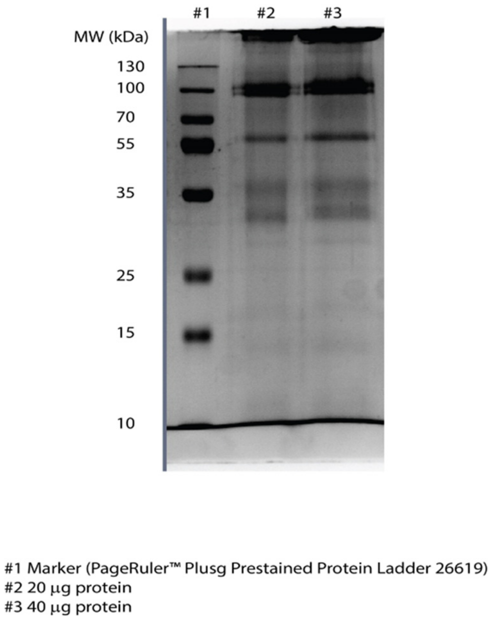

Figure S4. SDS-PAGE analysis result of the egg gel sample.
